# HPLC and ToF‒SIMS Analyses of *Toxicodendron vernicifluum* Tree Sap Mixed with Other Natural Lacquers

**DOI:** 10.3390/molecules26020434

**Published:** 2021-01-15

**Authors:** Hye Hyun Yu, Seung Wook Ham, Yeonhee Lee

**Affiliations:** 1 Advanced Analysis Center, Korea Institute of Science and Technology, Seoul 02792, Korea; 091615@kist.re.kr; 2 Department of Chemistry, Chung-Ang University, Seoul 06974, Korea; swham@cau.ac.kr

**Keywords:** urushiol, laccol, thitsiol, HPLC, ToF-SIMS

## Abstract

Lacquer sap has been used by humans from antiquitywhen it was treated as a luxury item because of its desirable physical properties. In modern times, although access barriers are lower, lacquer is still considered to be rare and valuable. Thus, low quality, inexpensive Vietnamese and Myanmarese lacquers and cashew nutshell liquid are frequently added to the costly *Toxicodendron vernicifluum* lacquer sap from Korea, China, and Japan. However, these blended lacquers can diminish the quality of artisan works. The *Toxicodendron vernicifluum* lacquer saps mixed with other natural lacquers were characterized using time-of-flight secondary-ion mass spectrometry (ToF−SIMS) and high-performance liquid chromatography (HPLC). ToF-SIMS provided the chemical structure of the lacquer monomer, copolymerized dimers, trimers, etc. HPLC provided quantitative analysis of the components of a randomly mixed lacquer. These techniques can be used to control the quality of commercial lacquer sap for the Asian lacquer industry and the traditional conservation of ancient objects.

## 1. Introduction

Lacquer sap has been used as a natural adhesive and coating material for wooden, ceramic, leather, and metal objects for thousands of years [1]. Lacquers were typically used for the preservation of household items and tools in Asian countries and are still used in the production of handicrafts and luxuries [2,3,4]. Lacquer was also used in Kanpou medicines because of its specific function, anti-oxidation ability, and anti-inflammatory ability. Usable lacquer sap was collected from six different types of *Anacardiaceae*, and, more frequently, from three types of trees: *Toxicodendron vernicifluum* (*T. vernicifluum*) from Korea, China, and Japan; *Toxicodendron succedaneum* (*T. succedaneum*) from North Vietnam and Taiwan; and *Gluta usitata* (*G. usitata*) from Myanmar and Thailand [5,6,7]. The main components of lacquer are polyphenols, but the functional groups can differ depending on the species and origins of the lacquer. The main component of *T. vernicifluum* is a 3-pentadecyl catechol with a side chain containing 0–3 double bonds. The common name for this compound is urushiol. Laccol from *T. succedaneum* is 3-heptadecyl catechol with a side chain that has 0–3 double bonds. Lastly, thitsiol from *G. usitata* is a catechol with a heptadecyl side chain or a phenyldodecyl at the meta or para position [8]. During the drying process, these major components are cross-linked to form a highly polymerized structure with oxygen and an oxidation enzyme, laccase. The completely dried lacquer films possess useful physical properties such as durability, thermal resistance, chemical resistance, moisture resistance, insulating properties, and the ability to produce high glosses and deter moths [9,10]. Because of these superb properties, which exceed those of any other obtainable natural material, lacquers from sap have been used since ancient times.

While usable lacquer sap can be extracted from the wounded bark tissues of typical trees, in the case of the cashew tree (*Anacardium occidentale* (*A. occidenale*)), a liquid with similar properties to lacquer sap can be derived from the shell of the cashew seed. *A. occidentale* usually grows in tropical countries, such as Central America, Brazil, and the Caribbean Islands. The major components of cashew nutshell liquid (CNSL) include anacardic acid, which is a hydroxybenzoic acid with a 15 or 17 carbon alkyl chain; cardol, which is resorcinol with a 0–3 double bond pentadecyl side chain at the meta position; and cardanol, which is a phenol with a pentadecyl side chain with 0–3 double bonds at the meta position [11,12]. Recently, CNSL has been chemically reformed for use in synthetic paint to achieve high production volumes at a low cost. Cashew paint is obtained from a condensation reaction with formaldehyde and melamine under acidic or alkaline conditions [13]. However, this reaction also produces formalin, which can release a hazardous gas upon drying.

While there are a variety of lacquers that can be used in paint, the quality and characteristics can vary. *T. vernicifluum* lacquer sap is considered to be of high quality owing to its high urushiol content; however, low production volumes make the lacquers expensive. Consequently, *T. vernicifluum* lacquer sap is sometimes mixed with cheaper, lower quality lacquer sap from different sources, such as *T. succedaneum* or *G. usitata* lacquer sap, or even with CNSL from *A. occidentale*. For this reason, analytical techniques that can identify the origin of lacquer sap and its contaminants have become necessary to control the quality of commercially available lacquer given its importance in cultural artwork preservation and domestic lacquer craft production.

Other research groups have investigated lacquer using various analytical techniques, such as X-ray photoelectron spectroscopy (XPS), Fourier transform infrared spectroscopy (FT–IR), high-performance liquid chromatography (HPLC), gas chromatography–mass spectrometry (GC–MS), and nuclear magnetic resonance spectroscopy [14,15,16,17]. Some of these studies have focused on investigating molecular composition and structural characteristics but do not provide quantitative results. Among these techniques, HPLC is employed most often and is the most accessible technique for identifying the constituents and quantifying the lacquer sap. The advantage of this technique is that it provides high separation, as well as adequate sensitivity and selectivity. Considering the concentration of the samples and the intensity of the results, HPLC should also be a suitable technique to quantify the components of the lacquer saps [18]. In addition, TOF–SIMS analysis can provide structural characterization of highly polymerized substances and relative quantitative information can be obtained from the intensities [19,20]. The analysis time is shorter than for HPLC, and mass spectrometry is a highly sensitive technique. In addition, it requires a minimal sample, and the solid can be measured without sample pretreatment [21].

The main purpose of this study was to characterize and quantify the components of the mixed lacquer sap product using reverse-phase HPLC. This will enable us to identify the origin of the lacquer saps and quantify the constituents. Additionally, ToF–SIMS was used to confirm the components of each polymeric lacquer film.

## 2. Results and Discussion

### 2.1. ToF–SIMS Compositional Analysis of Mixed Lacquers

**Figure 1:**
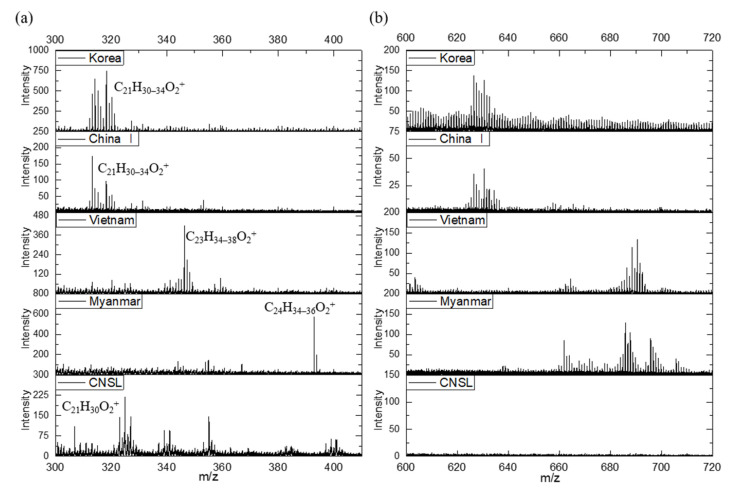
The positive ToF‒SIMS spectra of different lacquer saps: (**a**) *m*/*z* 300‒410; and (**b**) *m*/*z* 600‒720.

ToF–SIMS can be used to obtain chemical structure and molecular information from the surface layer of organic and polymeric materials [19,20,22]. The lacquer films from Korean and Chinese I *T. vernicifluum*, Vietnamese *T. succedaneum*, Myanmarese *G. usitata*, and Dongbang CNSL were prepared by solution casting of natural lacquer saps and characterized using ToF–SIMS. The positive ion ToF–SIMS spectra of various lacquer films were obtained using a Bi_3_^+^ primary ion beam under static SIMS conditions, as shown in Figure 1. The ToF–SIMS spectra of Korean and Chinese *T. vernicifluum* indicated the presence of urushiol lipids in the mass range *m*/*z* 300–410 and 600–720. Figures S1 and S2 show the positive and negative ion spectra of different lacquer saps on the extra mass range *m*/*z* 411–599. The major components of Korean and Chinese I lacquers were found to be pentadecyl catechols substituted in the 3-position by C15 chains with one, two, and three double bonds. They were observed at *m*/*z* 318.2 (C_21_H_34_O_2_^+^), 316.2 (C_21_H_32_O_2_^+^), and 314.2 (C_21_H_30_O_2_^+^). The ToF−SIMS spectra of the Vietnamese *T. succedaneum* and the Myanmarese *G. usitata* had peaks representing the main components of laccol (C_23_H_34_O_2_^+^, C_23_H_36_O_2_^+^, and C_23_H_38_O_2_^+^) and thitsiol (C_24_H_34_O_2_^+^, C_24_H_36_O_2_^+^, and C_24_H_38_O_2_^+^), respectively. The CNSL films produced spectra with high intensities representing the silicon-containing ions of poly(dimethylsiloxane) and cardol, which were determined to be the major components of CNSL. Urushiol, laccol, and thitsiol dimers were also found on the mass range *m*/*z* 600–720.

**Figure 2:**
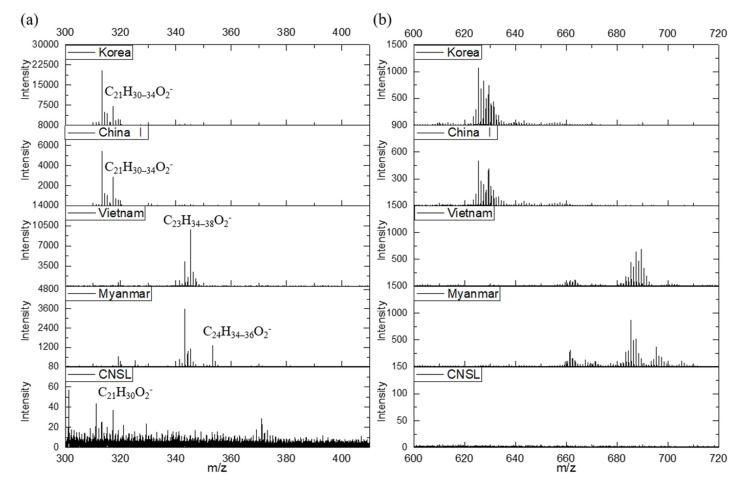
The negative ToF‒SIMS spectra of different lacquer saps: (**a**) *m*/*z* 300‒410; and (**b**) *m*/*z* 600‒720.

Figure 2 shows the negative ion ToF−SIMS spectra for Korean and Chinese I *T. vernicifluum*, Vietnamese *T. succedaneum*, Myanmarese *G. usitata*, and CNSL in the mass ranges *m*/*z* 300–410 and 600–720. We observed specific peaks for 3-pentadecyl catechols from Korean and Chinese I, 3-heptadecyl catechols from Vietnamese, and 4-heptadecenyl catechols and 3-phenyldodecyl catechols from Myanmarese lacquers. We also observed urushiol, laccol, and thitsiol dimers with two repeating units of its main component in the mass range *m*/*z* 600–720. Therefore, ToF-SIMS is an important technique to identify and discriminate the main components of different natural lacquers because it provides the detailed information on the molecular and fragment ions as well as elemental ions.

**Figure 3:**
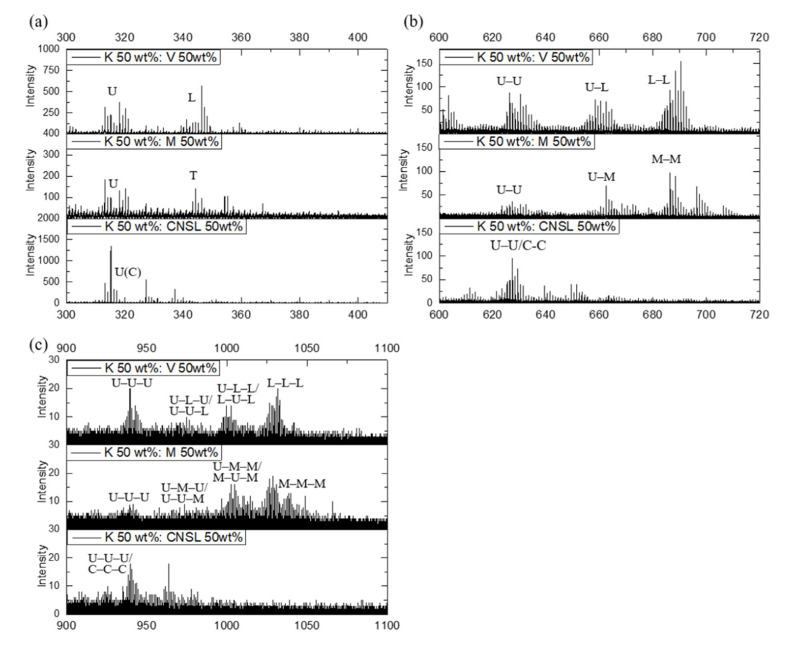
The positive Korean‒Vietnamese, Korean‒Myanmarese, and Korean‒CNSL mixed lacquer spectra: (**a**) *m*/*z* 300‒410; (**b**) *m*/*z* 600‒720; and (**c**) *m*/*z* 900‒1100.

Figure 3 shows the ToF-SIMS spectra for the 50:50 wt% mixtures of Korean *T. vernicifluum* lacquer with Vietnamese *T. succedaneum*, Myanmarese *G. usitata*, and *A. occidentale* from CNSL. The rest mass ranges *m*/*z* 411–599 and 721–899 are shown in Figures S3 and S4. The ToF−SIMS spectra for Korean and Vietnamese mixed lacquer indicated the presence of urushiol−laccol dimers as well as pure urushiol−urushiol and laccol–laccol dimers. The ToF–SIMS spectra for the Korean and Myanmarese mixture also indicated the presence of urushiol−urushiol, thitsiol−thitsiol, and urushiol–thitsiol dimers, which were generated by the cross-coupling of catechol radicals. In the high-mass range (*m*/*z* 900–1100), the ToF–SIMS spectra of Korean–Vietnamese (Korean–Myanmarese) mixed lacquer films indicated the presence of urushiol–laccol–urushiol (U–L–U) [urushiol–thitsiol–urushiol (U–T–U)] and laccol–urushiol–laccol (L–U–L) [thitsiol–urushiol–thitsiol (T–U–T)] trimers. The polymer formation mechanism in the mixed lacquers can be explained as the laccase-catalyzed dehydrogenation of urushiol, radical formation of laccol or thitsiol by a radical transfer reaction, and then a coupling reaction between two radical species to produce dimers. These dimers further generate radical species and couple among themselves to give oligomers such as trimer, tetramer, etc.

**Figure 4:**
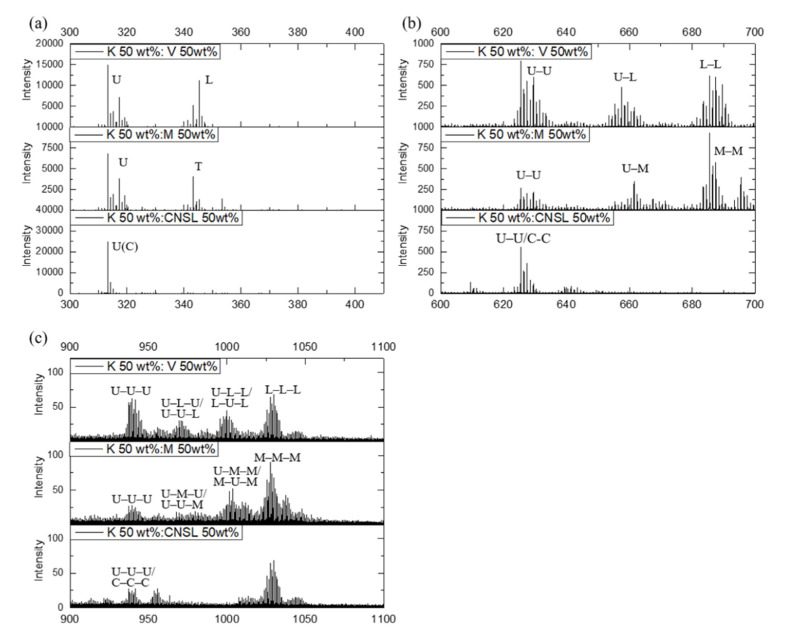
The negative Korean‒Vietnamese, Korean‒Myanmarese, and Korean‒CNSL mixed lacquer spectra: (**a**) *m*/*z* 300‒410; (**b**) *m*/*z* 600‒720; and (**c**) *m*/*z* 900‒1100.

We also observed the monomer, dimer, and trimer of the main lipid catechols in the negative ion ToF–SIMS spectra for the mixed lacquers, as shown in Figure 4. These ToF–SIMS results indicate that urushiol, laccol, and thitsiol from different lacquer trees can be copolymerized by the radical transfer of a laccase-catalyzed oxidation.

### 2.2. HPLC Quantitative Analysis of Mixed Lacquers

**Figure 5:**
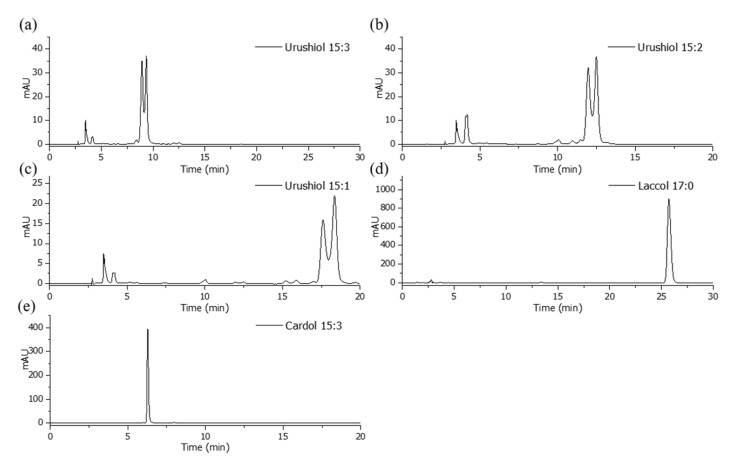
The HPLC chromatograms of standard samples: urushiol 15:3 (**a**); urushiol 15:2 (**b**); urushiol 15:1 (**c**); laccol 17:0 (**d**); and cardol 15:3 (**e**).

HPLC can be used to calculate the quantities of different components of lacquer sap by separating the complex chemical components included in the lacquers. To identify the main components of the raw lacquer saps from Korea, China I, Japan, Vietnam, Myanmar, and cashew trees, the standards were first analyzed by HPLC (Figure 5). The purchased compounds, urushiol 15:3, urushiol 15:2, urushiol 15:1, laccol 17:0, and cardol 15:3, were used as standards which help to differentiate the three species: *T. vernicifluum*, *T. succedanea*, and *A. occidentale*. The chromatograms confirm the high purity of the standards and show high separation due to the differences in polarity of the standard compounds and columns. The major peaks produced by urushiol 15:3 can be identified at 8.92 and 9.30 min, and urushiol 15:2 produced major peaks at 11.95 and 12.48 min. The major peaks for urushiol 15:1 were observed at 17.63 and 18.38 min. Laccol 17:0 is the only standard that was analyzed using a different solvent system, and its principal peak is present at 25.76 min. Cardol 15:3 had a retention time of 8.31 min and was detected using a wavelength of 280 nm. The retention time, response factor, limit of detection, and limit of quantification, which were derived from the standard chromatograms, are represented at Table 1. The limit of detection (LOD) and quantification (LOQ) were determined as the standards concentration that produced signal-to-noise ratio larger than 3 and 10, respectively. 

**Figure 6:**
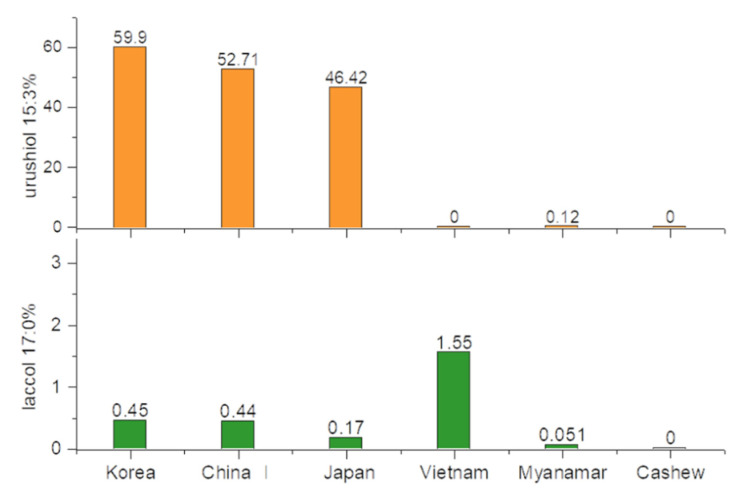
The urushiol 15:3 and laccol 17:0 contents in lacquer from different producers.

The peaks of the standard samples were used to identify and then quantify the main components of the different types of lacquer saps. The proportions of urushiol 15:3 and laccol 17:0 in Korean, Chinese I, Japanese, Vietnamese, and Myanmarese lacquers and CNSL are shown in Figure 6. The Korean, Chinese I, and Japanese chromatograms indicate greater concentrations of urushiol 15:3 than other lacquer saps, which indicates that they contain *T. vernicifluum* lacquer. In contrast, the chromatogram for *T. succedanea* Vietnamese lacquer indicates a relatively high quantity of laccol 17:0 in the analyte, suggesting that this was one of the main components. The main components of Vietnamese lacquer with high contents are 3-heptadecylcatechol with a side chain containing one, two, and three double bonds. However, these catechols were not used as a standard because they were not available commercially.

**Figure 7:**
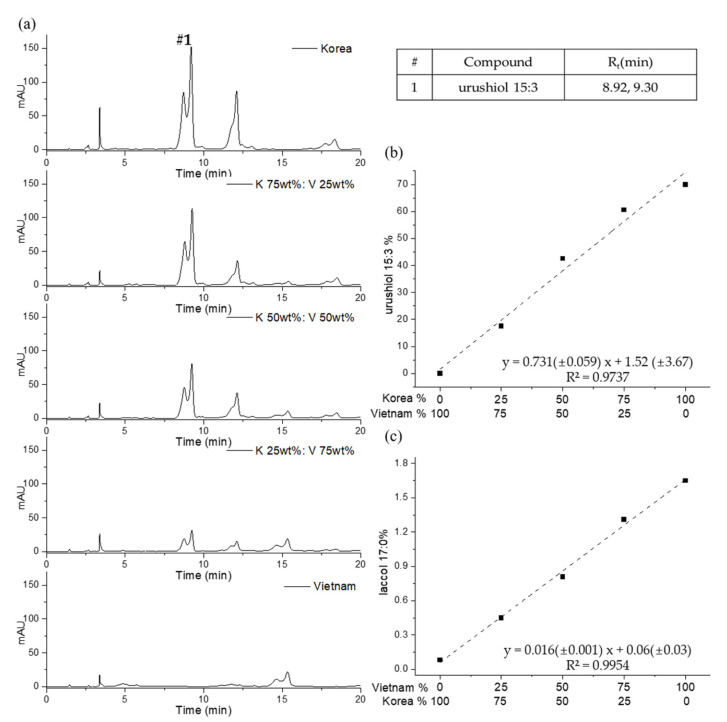
The HPLC chromatograms of Korean‒Vietnamese mixed lacquer sap (**a**); and the quantitative calibration curves of urushiol 15:3 (**b**) and laccol 17:0 (**c**).

The lacquers from different producers were blended with various ratios to draw a calibration curve by quantifying the amounts of main components for estimation of the unknown lacquer sap. Figure 7a shows the chromatograms of various proportions of Korean (*T. vernicifluum*) and Vietnamese (*T. Succedaneum*) lacquer (0%, 25%, 50%, 75%, and 100%), analyzed using the urushiol experimental conditions. The major urushiol 15:3 peak is distinctive in the chromatogram for Korean lacquer sap, while the urushiol 15:3 peak for the Vietnamese lacquer is indistinguishable from the baseline noise. The other chromatograms for various blended mixtures featured remarkable variations in the intensity of the urushiol 15:3 peaks due to varying concentrations of Korean lacquer sap in the samples. The quantity of each lacquer type was calculated using the peak area of the standards as a reference. The calibration curve is shown in Figure 7b. The urushiol calibration curve has a slope of 0.731, an intercept of 1.52, and a coefficient of determination (R^2^) of 0.9737, where x is the concentration of Korean lacquer. Additionally, the laccol 17:0 constituents in mixed lacquer were measured using the laccol measurement conditions; the calibration curve equation was y = 0.016x + 0.06 and the coefficient of determination was 0.9954 with the x-axis displaying the concentration of Vietnamese lacquer (Figure 7c).

**Figure 8:**
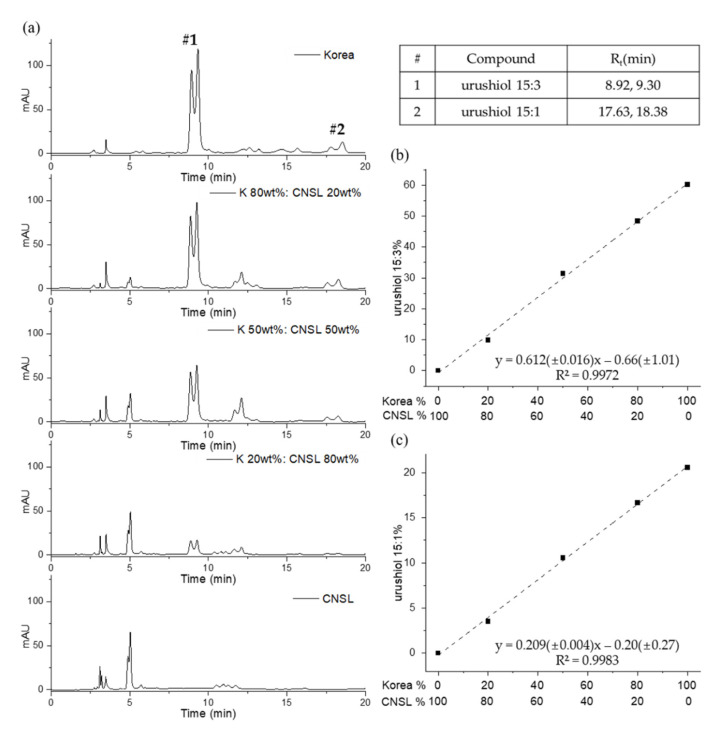
The HPLC chromatograms of Korean‒CNSL mixed lacquer sap (**a**); and the quantitative calibration curves of urushiol 15:3 (**b**) and urushiol 15:1 (**c**).

The Korean lacquer sap and CNSL (from *A. occidentale*) were blended with the proportions 0%, 20%, 50%, 80%, and 100% and analyzed with the urushiol measurement conditions (Figure 8). The chromatograms were compared with those for the urushiol 15:3 and urushiol 15:1 standards to determine the Korean lacquer composition. The intensity of the urushiol 15:3 and 15:1 peaks gradually diminished following the proportion of the Korean lacquer components. Calibration curves were generated from different concentrations of Korean lacquer sap with peaks representing urushiol 15:3 and urushiol 15:1. These calibration curves have high coefficients of determination (0.9972 for urushiol 15:3 and 0.9983 for urushiol 15:1), which indicate the accuracy of the calculated quantities for both urushiol 15:3 and urushiol 15:1.

**Figure 9:**
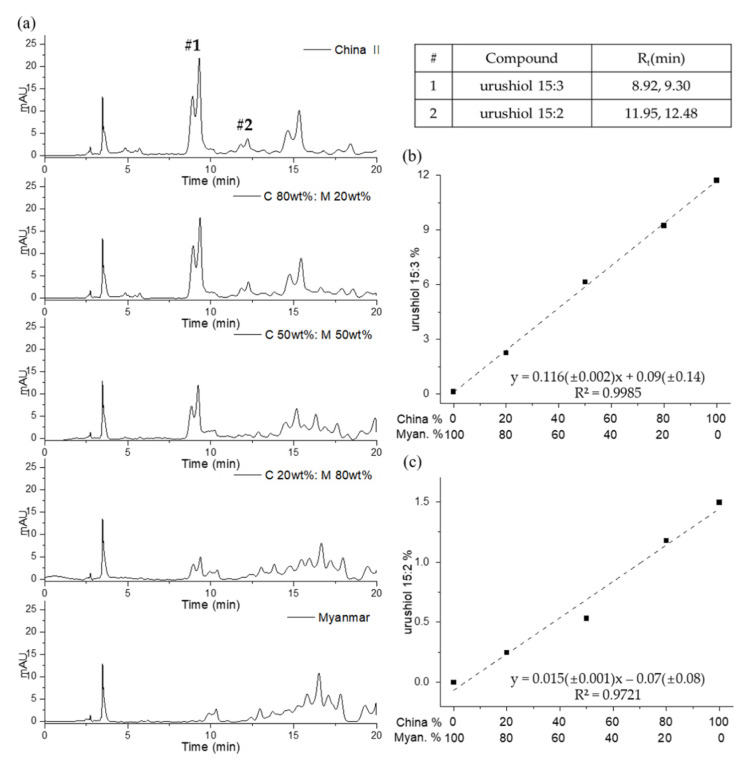
The HPLC chromatograms of Chinese Ⅱ‒Myanmarese mixed lacquer sap (**a**); and the quantitative calibration curves of urushiol 15:3 (**b**) and urushiol 15:2 (**c**).

The Chinese II and Myanmarese lacquer sap were blended in the proportions 0%, 20%, 50%, 80%, and 100% and analyzed using the urushiol method to determine the urushiol 15:3 and urushiol 15:2 component in the mixed lacquer. The urushiol content in Chinese II sap is different from the contents of Chinese I, which is indicated in Figure 6. They were obtained from the same vendor, but, because of the time lag between purchases, different types of lacquer sap from different sources may have been used, which caused variations in the urushiol content. The HPLC chromatograms indicate variations in intensity for urushiol 15:3 and urushiol 15:2 due to the concentrations of mixed Chinese II and Myanmarese lacquer sap (Figure 9). The quantities were calculated from the area of the major peaks representing urushiol 15:3 and urushiol 15:2, and the calibration curves are displayed with the concentration of Chinese II lacquer on the x-axis. The value of the coefficient of determination (R^2^) was 0.9985 for urushiol 15:3 and 0.9721 for urushiol 15:2.

The Korean‒Vietnamese, Korean‒CNSL, and Chinese II‒Myanmarese blended lacquer sap were mixed with different proportions to make calibration curves, and each urushiol was used differently to perform quantification because of the overlapping with other peaks from the original lacquer sap. The urushiol 15:2 peaks are duplicated with CNSL peaks, resulting in the highest height at 50:50 ratio. Similarly, the urushiol 15:1 peaks coincide with the main peaks of Myanmarese lacquer. All the calibration curves derived from main components had high coefficients of determination (>0.97), indicating the accuracy of the quantitative results with urushiol 15:3, urushiol 15:2, urushiol 15:1, and laccol 17:0. Especially, the well resolved peaks with high intensity showed relatively high values of the coefficients of determination (>0.99). The reliability of mixed lacquer calibration curve was secured with high linearity and reconfirmation by two different main components.

**Figure 10:**
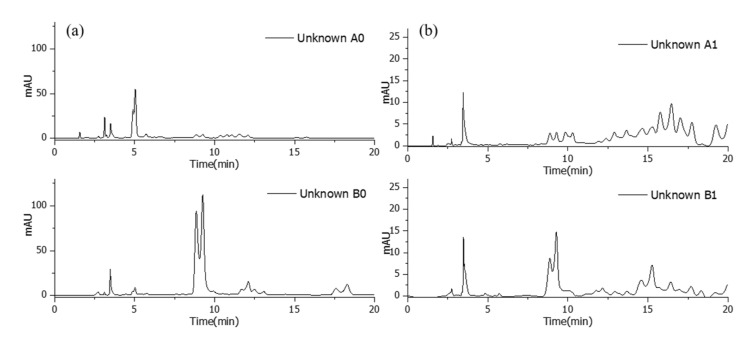
The chromatograms of unknown ratio mixed lacquer for blind test: (**a**) Korean‒CNSL; and (**b**) Chinese Ⅱ‒Myanmarese.

With these obtained calibration curves, the quantity of unknown lacquer sap can be estimated by the mixed types of the lacquer. Additionally, a blind test was performed by mixing the Korean lacquer and the CNSL in the ratios of 10:90, and 90:10, as well as the Chinese II and Myanmarese lacquer sap in the ratios of 10:90 and 70:30. The mixed lacquer saps were analyzed with urushiol measurement conditions as in the preceding HPLC analysis, and the results are shown in Figure 10. The urushiol 15:3 and urushiol 15:1 concentrations were calculated using the Korean-CNSL calibration equation, and the Chinese II–Myanmarese calibration equation was applied to calculate the urushiol 15:3 and urushiol 15:2 contents. The results are summarized in Table 2. Quantitation results show ±3.9% as the highest value of error and ±0.1% as the lowest value of error compared with the actually used contents of lacquer sap. The unknown mixed lacquer sap could be quantified precisely using standards. We also used additional standard samples to confirm the results and obtain more accurate and convincing outcomes.

## 3. Materials and Methods

### 3.1. Materials

Lacquer sap from different producers was used on its own or mixed to generate a calibration curve for the quantitative analysis. The Korean lacquer was provided by a human cultural asset, Hyngman Lee, and was collected in Wonju city, Korea. The Japanese, Chinese I and II, and Myanmarese lacquer saps were purchased from the local market, Pyung Hwa Shell (Seoul, Korea). The two Chinese lacquers I and II were purchased at different times, which resulted in the two samples having different properties. The Vietnamese lacquer was originally obtained from *T. succedaneum* and provided by the Vietnam–Korea Institute of Science and Technology (Hanoi, Vietnam). Additionally, Dongbang cashew, which CNSL commonly includes, was purchased from Dongbang Cashewchil Inc. (Jincheon, Korea). The mixed lacquer was prepared by mixing *T. succedaneum*, *G. usitata*, or *A. occidentale* with *T. vernicifluum* lacquer sap homogeneously with different proportions, as shown in Table 3. For fluid samples, organic solvents such as chloroform and methanol (Sigma-Aldrich Co., St. Louis, MO, USA) were used to prepare the sample for HPLC analysis. Turpentine oil (Wholeart, Seoul, Korea) was used to make a film-type sample for ToF-SIMS analysis.

The chemical structures and formulae of the standard samples that were used to distinguish the main component and quantify the lacquer sap mixed ratio are listed in Table 4. 3-(8Z,11Z,14-pentadecatrienyl)-1,2-benzenediol (urushiol 15:3), 3-(8Z,11Z-pentadecadienyl)-1,2-benzenediol (urushiol 15:2), and 3-(8Z-pentadecenyl)-1, 2-benzenediol (urushiol 15:1) were obtained from PhytoLab GmbH & Co. KG (Vestenbergsgreuth, Germany). 3- heptadecylcatechol (laccol 17:0) was obtained from BOC Sciences (Shirley, NY, USA). 5-(8Z,11Z)-8,11,14-pentadecatrienyl-1,3-benzenediol (cardol 15:3) was purchased from Angene International Limited (London, UK).

### 3.2. Methods

#### 3.2.1. Time-of-Flight Secondary Ion Mass Spectrometry

The mixed lacquer sap was blended with turpentine oil with a volumetric ratio of 1:1 and coated on the Si wafer with a blade with approximately 10 μm thickness. The samples were then dried in a humidity-controlled chamber with 80% relative humidity at 22 °C for 2 h. The film-type samples were analyzed using positive and negative mode ToF–SIMS 5 (ION-TOF GmbH, Münster, Germany) with a base pressure of 4.0 × 10^−8^ Pa [23]. The source was a pulsed 30 keV Bi_3_^+^ beam, and the target current was maintained at 0.3 pA. A cycle time of 150 μs was used for the mass range of *m*/*z* 0–1870 and ion doses lower than 10^13^ ions/cm^2^, which is the static SIMS limit. The mass spectra for the lacquer films were collected from an area of 100 × 100 µm^2^ and were used to distinguish different types of lacquers. The ToF–SIMS analysis was carried out for more than three points for each sample to ensure repeatability and to obtain representative spectra.

#### 3.2.2. High-Performance Liquid Chromatography

HPLC analysis was carried out using an Agilent Technologies 1290 infinity instrument (Agilent Technologies, Inc., Santa Clara, CA, USA). The separation was conducted with a C18 reverse-phase column (YMC-Pack Pro 4.6 mm I.D. S-5 μm, 12 nm). Individualized methods were designed for the detection of urushiols, laccol, and cardol, and they are organized in Table S1. The samples were diluted differently according to the main components being analyzed. For urushiol analysis, the lacquer sap was prepared at a concentration of 0.5 wt% by dissolving in chloroform and for laccol analysis, the sap was dissolved in methanol to obtain a concentration of 2.5 wt%. These samples were vortexed for 5 min and centrifuged for approximately 10 min. The extracted supernatant was filtered through a 0.45 μm polytetrafluoroethylene membrane filter. Ten microliters of each prepared sample were injected into the HPLC. To analyze the three types of urushiols and cardol, we used a column temperature of 30 °C, an isocratic mobile phase of acetonitrile/0.1% trifluoroacetic acid in dilute water (90:10, *v*/*v*), and a flow rate of 1 mL/min [24]. The mobile phase had little effect on the separation of lacquer samples. For the laccol, we used a column temperature of 40 °C with a mobile phase of acetonitrile/0.1% tetrahydrofuran in dilute water (95:5, *v*/*v*) at a constant flow rate of 1 mL/min as above. Three different ultraviolet wavelengths were selected for the detection and quantification of the lacquer samples (260 nm for urushiols, 280 nm for cardol, and 210 nm for laccol). The measurements were repeated to confirm the results.

## 4. Conclusions

In this study, ToF‒SIMS was used to characterize the main components of different lacquer saps, such as urushiol in *T. vernicifluum*, laccol in *T. succedaneum*, thitsiol in *G. Usitata*, and cardol in *A. occidentale* trees. Additionally, we observed the copolymerization of characteristic constituents of blended lacquers that occurs by the cross-coupling reaction of catechol radicals from urushiol–laccol, urushiol–thitsiol, and urushiol–cardol. Moreover, the quantitative analysis for two different mixed lacquers was carried out using HPLC. Catechol standards such as urushiol 15:3, urushiol 15:2, urushiol 15:1, and laccol 17:0 were used to conduct the comparative analysis and obtain calibration curves. The contents of mixed lacquer sap were analyzed by comparing with the calibration curves of Korean‒Vietnamese, Korean‒CNSL, and Chinese II‒Myanmarese mixed lacquer sap. The unknown mixed lacquer could be estimated using the concentration of *T. vernicifluum* lacquer. This research will be used to grade the quality of commercially distributed lacquer sap to ensure it meets the physical specifications for handicraft and cultural heritage preservation.

## Figures and Tables

**Table 1:** The retention time, response factor, limit of detection (LOD), and limit of quantification (LOQ) of standard samples.

Compound	Retention Time (Rt, min)	Response Factor (R_f_*10^−3^)	LOD (μg/mL)	LOQ (μg/mL)
urushiol 15:3	8.92, 9.30	1.06	0.55	1.86
urushiol 15:2	11.95, 12.48	1.23	0.57	1.90
urushiol 15:1	17.63, 18.38	1.77	0.52	1.73
laccol 17:0	25.76	2.78	0.08	0.26
cardol 15:3	8.30	2.62	0.08	0.25

**Table 2:** The HPLC quantitative analysis results of unknown lacquer blind test.

**Korean‒CNSL**	**CNSL Composition (wt%) [±SD]**		
	**Urushiol 15:3**	**Urushiol 15:1**	**Used Contents (wt%**)
Unknown A0	93.3 (±1.4)	93.9 (±1.0)	90.0
Unknown B0	8.8 (±1.3)	10.0 (±1.0)	10.0
**Chinese** **II** **‒Myanmarese**	**Myanmarese Composition (wt%) [±SD]**		
	**Urushiol 15:3**	**Urushiol 15:2**	**Used Contents (wt%)**
Unknown A1	89.9 (±0.8)	86.2 (±4.8)	90.0
Unknown B1	33.0 (±0.4)	31.7 (±2.2)	30.0

**Table 3:** The list of the proportions of mixed lacquer samples.

Blend	Korean (wt%)	Vietnamese (wt%)	Blend	Korean (wt%)	CNSL (wt%)	Blend	Chinese II (wt%)	Myanmarese (wt%)
KV01	0	100	KC01	0	100	CM01	0	100
KV02	25	75	KC02	20	80	CM02	20	80
KV03	50	50	KC03	50	50	CM03	50	50
KV04	75	25	KC04	80	20	CM04	80	20
KV05	100	0	KC05	100	0	CM05	100	0

**Table 4:** The chemical formulae and structures of the standard samples.

**urushiol 15:3**	3-[(8ZE,11Z,14E)-pentadeca-8,11,14-trienyl])- 1,2-benzenediol C_21_H_30_O_2_ M.W. 314.47	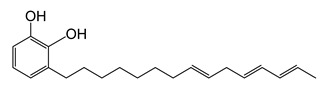
**urushiol 15:2**	3-(8Z,11Z-Pentadecadienyl)-1,2-benzenediol C_21_H_32_O_2_ M.W. 316.49	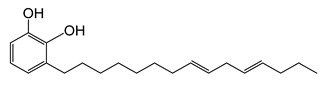
**urushiol 15:1**	3-(8Z-Pentadecenyl)- 1,2-benzenediol C_21_H_34_O_2_ M.W. 318.49	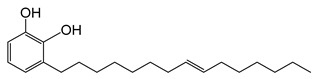
**laccol 17:0**	3-heptadecylbenzene-1,2-diol C_23_H_40_O_2_ M.W. 348.57	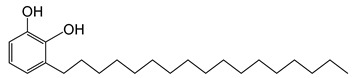
**cardol 15:3**	5-(8Z,11Z)-8,11,14-Pentadecatrien-1-yl-1,3-benzenediol C_21_H_30_O_2_ M.W. 314.47	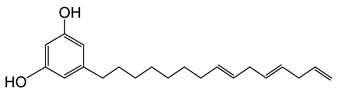

## Data Availability

The data presented in this study is contained within this article or supplementary material.

## Supplementary Materials

The following are available online at, Figure S1: The positive ToF‒SIMS spectra of different lacquer saps at *m*/*z* 411‒599, Figure S2: The negative ToF‒SIMS spectra of different lacquer saps at *m*/*z* 411‒599, Figure S3: The positive Korean‒Vietnamese, Korean‒Myanmarese, and Korean‒CNSL mixed lacquer spectra: (a) *m*/*z* 411‒599; and (b) *m*/*z* 721‒899. Figure S4: The negative Korean‒Vietnamese, Korean‒Myanmarese, and Korean‒CNSL mixed lacquer spectra: (a) *m*/*z* 411‒599; and (b) *m*/*z* 721‒899. Table S1: HPLC conditions for urushiol 15:3, urushiol 15:2, urushiol 15:1, laccol 17:0, and cardol 15:3 standard samples.
