# Supplementary material for: HPLC and ToF‒SIMS Analyses of Toxicodendron vernicifluum Tree Sap Mixed with Other Natural Lacquers
Source: Molecules. 2021 Jan 15;26(2):434. doi: 10.3390/molecules26020434 (PMC7830298; doi:10.3390/molecules26020434)
Supplement: Supplementary file 1 [file molecules-26-00434-s001.pdf]

Supplementary Materials

HPLC and ToF–SIMS Analyses of *Toxicodendron vernicifluum* Tree Sap Mixed with Other Natural Lacquers

Hye Hyun Yu[1,2], Seung Wook Ham[2] and Yeonhee Lee[1*]

[1]Advanced Analysis Center, Korea Institute of Science and Technology, Seoul 02792, Korea
[2]Department of Chemistry, Chung-Ang University, Seoul 06974, Korea
* Correspondence: author; E-mail: yhlee@kist.re.kr, Tel: +82-2-958-5971, FAX: +82-2-958-5969

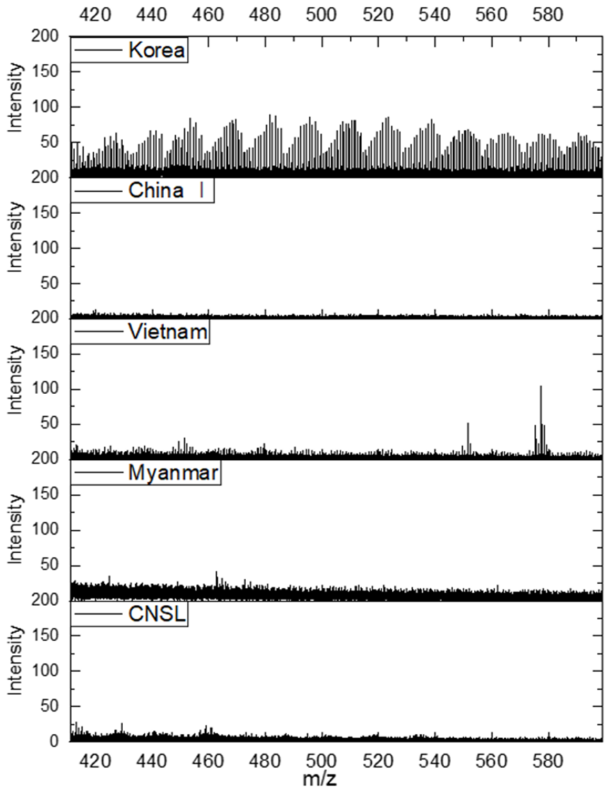

Figure S1. The positive ToF–SIMS spectra of different lacquer saps: *m/z* 411–599.

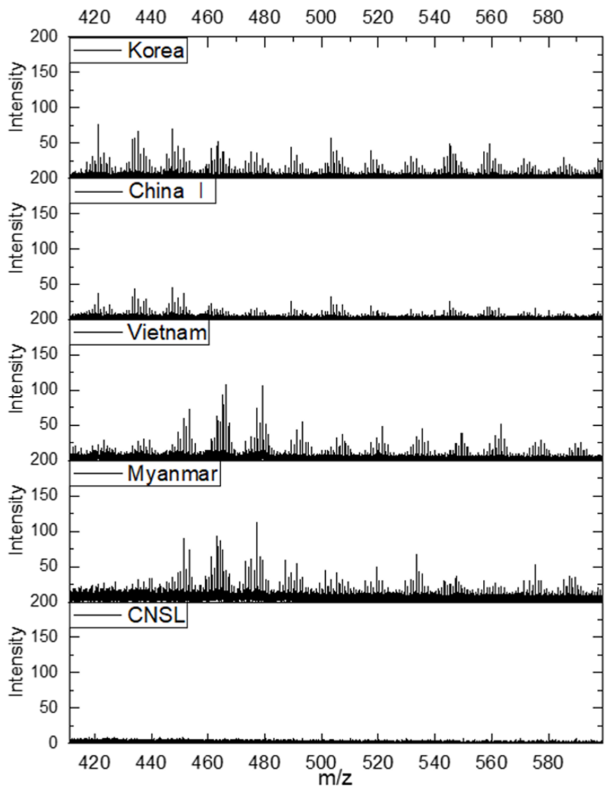

Figure S2. The negative ToF–SIMS spectra of different lacquer saps: *m/z* 411–599.

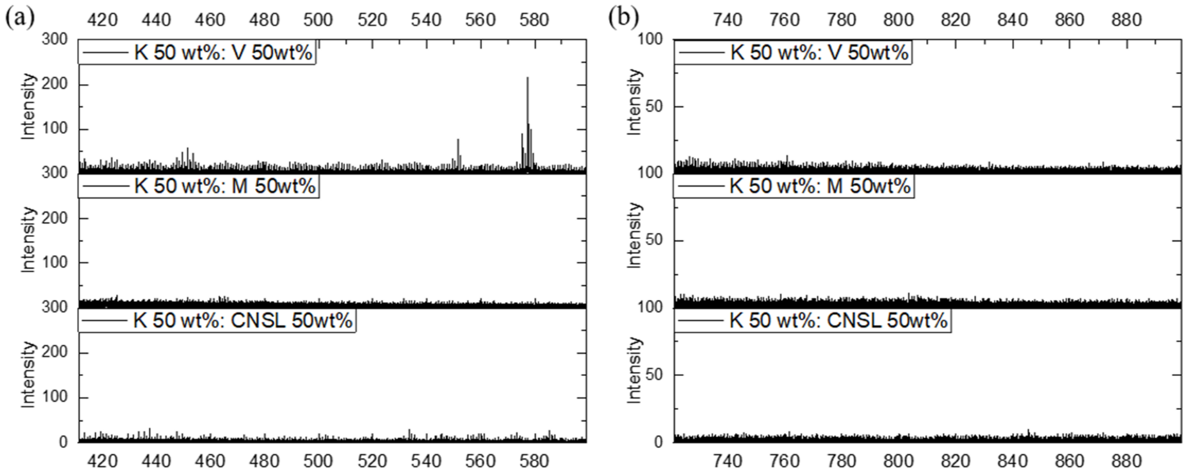

Figure S3. The positive Korean–Vietnamese, Korean–Myanmarese, and Korean–CNSL mixed lacquer spectra: (a) *m/z* 411–599; and (b) *m/z* 721–899.

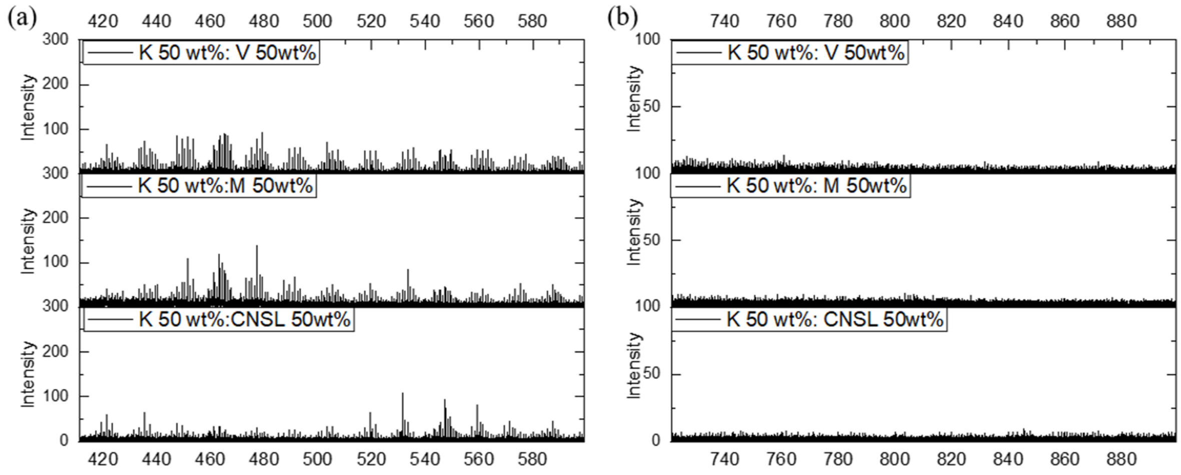

Figure S4. The negative Korean–Vietnamese, Korean–Myanmarese, and Korean–CNSL mixed lacquer spectra: (a) *m*/*z* 411–599; and (b) *m*/*z* 721–899.

Table S1. HPLC conditions for urushiol 15:3, urushiol 15:2, urushiol 15:1, laccol 17:0, and cardol 15:3 standard samples.

	**urushiols (urushiol 15:3, 15:2, 15:1)**	**laccol 17:0**	**cardol 15:3**
Column	C18 reverse-phase column<br>YMC-Pack Pro C18, 250 × 4.6 mm I.D. S-5 μm, 12 nm		
Mobile phase	A: acetonitrile<br>B: acqueous trifluoroactic acid, 0.1 vol%<br>(A:B=90:10)	A: 0.1% THF in dilute water<br>B: acetonitrile<br>(A:B=95:5)	A: acetonitrile<br>B: acqueous trifluoroactic acid, 0.1 vol%<br>(A:B=90:10)
Flow rate	1 mL/min	1 mL/min	1 mL/min
Injection volume	10 μL	10 μL	10 μL
Temperature	30°C	40°C	30°C
Detection	UV, 260 nm	UV, 210 nm	UV, 280 nm
Sample solvent	chloroform	methanol	chloroform
